# The role of Angiogenesis and remodeling (AR) associated signature for predicting prognosis and clinical outcome of immunotherapy in pan-cancer

**DOI:** 10.3389/fimmu.2022.1033967

**Published:** 2022-11-21

**Authors:** Xiaojiao Sun, Zhuo Zhang, Zhiqi Wang, Ran Xie, Chuxiao Yi, Huiyu Liu, Xiaowei Chi, Tiancheng Li, Haitao Liu, Yi Han, Xiaocong Pang, Yimin Cui, Zhenming Liu

**Affiliations:** ^1^ State Key Laboratory of Natural and Biomimetic Drugs, School of Pharmaceutical Sciences, Peking University Health Science Center, Peking University, Beijing, China; ^2^ Department of Pharmacy, Peking University First Hospital, Beijing, China; ^3^ Institute of Clinical Pharmacology, Peking University, Beijing, China; ^4^ Departments of Otorhinolaryngology-Head and Neck Surgery, Peking University First Hospital, Beijing, China; ^5^ Department of Thoracic Surgery, Beijing Thoracic Hospital, Beijing, China

**Keywords:** angiogenesis, remodeling, tumor microenvironment, immunotherapy, pan-cancer

## Abstract

**Background:**

Angiogenesis and remodeling (AR) is necessary for the growth and metastasis of cancers. Although AR related genes involved in this process are reported, the correlation between AR and clinical outcome, immune cell infiltration, and immunotherapy is still unknown in diverse cancers. This study aimed to investigate the role of AR in the tumor immune microenvironment (TIME) in pan-cancer, and explore its values in prognostic prediction and therapeutic responses.

**Methods:**

Firstly, AR genes (including angiogenesis genes and blood vessel remodeling genes) are collected from MsigDB database. The differential expression, and prognostic value of AR genes were studied in 33 tumor types based on TCGA and GTEx data. The AR score of each sample was calculated using the “ssGSEA” function of R package “GSVA” in pan-cancer. The correlation of the AR score with TIME index, such as the amount of stromal and immune components and the immune cell infiltration, was evaluated *via* integrating multiple computational methods. And we also utilized IMvigor210 and GSE78220 data to explore the prediction value of the AR score on the immunotherapy response.

**Results:**

Significant differences in AR gene expression between tumors and adjacent normal tissues were found in most cancer types. The AR score varied depending on the types of tumors, and high score was related to worse survival in various tumors, such as pancreatic and stomach adenocarcinoma and so on. Moreover, the AR score was further explored to be positively correlated with proportions and pathways of immune and stromal in TIME. And the AR score was positively correlated with immunosuppressive cells, including TAMs and iTregs, while negatively with CD8+ T cells. Further analysis revealed that patients with high AR had worse therapy efficacy and survival status in bladder cancer and melanomas.

**Conclusions:**

Our systematic analysis revealed that AR is closely associated TIME, and prognosis, and clinical characteristics in multiple cancers. Targeting AR genes may activate immune microenvironment and increase the efficacy of immunotherapy.

## Introduction

Angiogenesis and remodeling (AR), which is considered to be one of the hallmarks of cancer, is required for tumor growth and metastasis (1–3). A lot of molecules have been identified to play a critical role in the modulation of AR, but vascular endothelial growth factor (VEGF) family and the angiopoietin (Ang 1-2)/Tie-2 pathway are the most studied (4). And some recent studies indicated that high levels of these angiogenic markers predict poor prognosis in various types of tumors (5–7). Therefore, anti-angiogenesis therapy attracted intensive attention and a variety of VEGF-VEGFR targeted drugs came out, following the first anti-angiogenesis agent, bevacizumab (8).

Immune tolerance is another normal physiologic mechanism that is hijacked by tumors. Nowadays, immune checkpoint inhibitors (ICIs) have made an indelible mark in the field of cancer treatment in the modern era, demonstrating a long-lasting clinical activity against many cancers (9). But the main challenge in the cancer immunotherapy field is moving immune checkpoint inhibitors (ICIs) activities to noninflamed tumors and overcoming resistance due to the immune-suppressive microenvironment (10). Actually, hyperactive AR factors, like VEGF and Ang-2 families, drive immune suppression by the proliferation and differentiation of activated immune effector cells, while recruiting suppressive tumor-associated immune cells, like regulatory T cells (Treg), myeloid-derived suppressor cells (MDSC), and tumor-associated macrophages (TAM) (11). Hence, anti-angiogenesis therapy not only prunes blood vessel, but also modulates the tumor immune microenvironment (TIME) (12). Accordingly, combination with anti-angiogenesis agents, is one of the many strategies currently under investigation to improve the effects of immunotherapies (13, 14).

Nowadays, an increasing number of clinical trials have begun to test the efficacy of ICIs/anti-angiogenesis combination to reverse the immune suppression-driven by AR (12). But the majority of promising data was generated in renal cell carcinoma (RCC), a cancer with both high angiogenic and immunogenic properties (10). Recently, Zhang, et al. found angiogenesis-associated genes accurately predicted the clinical outcome of angiogenesis-associated genes in gastric cancer patients and immunotherapeutic effect (15). However, it remains unclear whether this combination would be proven to be as effective in other tumor types. In-depth studies of AR and TIME interactions in the pan-cancer landscape might provide novel strategies for subsequent targeted immunotherapies. Herein, we first performed a pan-cancer systematic analysis of AR genes in 33 tumor types from The Cancer Genome Atlas (TCGA) and Genotype-Tissue Expression (GTEx), including the gene alteration, expression, clinical features, and prognostic values. Secondly, we calculated the AR score and explored the association of AR score with TIME and immunotherapy response, providing the indication of AR score on the efficacy of immunotherapy.

## Materials and methods

### Source of hallmark gene sets

Hallmark gene sets of AR were searched on the Molecular Signatures Database (MSigDB) (http://www.gsea-msigdb.org/gsea/msigdb/index.jsp), using the following keywords of “HALLMARK_ANGIOGENESIS” and “GOBP_BLOOD_VESSEL_REMODELING”.

### Data collection and pre-processing

The expression profiles, tumor somatic mutant profiles and clinical information of TCGA (https://portal.gdc.cancer.gov/) and GTEx (https://www.gtexportal.org/home/) were downloaded from the UCSC Xena (https://xenabrowser.net/datapages/) database. The RNA-seq data in transcripts per million read (TPM) format were analyzed and compared after log2 conversion. The immune cell infiltration data of TCGA were obtained from three different sources, including a supplementary information of a published work (16), ImmuCellAI database (17) (http://bioinfo.life.hust.edu.cn/ImmuCellAI#!/) and TIMER2 database (18) (http://timer.cistrome.org/). The microsatellite instability (MSI) data was obtained from the study by Bonneville (19), reporting the prevalence and extent of MSI across 39 cancers. The immunotherapy cohorts GSE78220 and IMvigor210 were respectively obtained from Gene Expression Omnibus (GEO) database (https://www.ncbi.nlm.nih.gov/gds/?term=GSE78220) and R package “IMvigor210CoreBiologies” (20).

### Gene enrichment analysis

Gene set variation analysis (GSVA) and gene set enrichment analysis (GSEA) were performed using R package “GSVA” and “clusterprofiler” to explore the associated functions of AR genes. The HALLMARK pathways in GSVA were downloaded from the MsigDB database (http://software.broadinstitute.org/gsea/msigdb/index.jsp).

### Prognostic analysis of the AR score

The ssGSEA function of R (version 4.1.1) package “GSVA” was used to calculate AR score of each patient in TCGA cohort. R package “survminer” and “survival” were used to perform the Univariate Cox regression (uniCox) and Kaplan-Meier analyses to explore the relationship of AR score with the survival of patients, including overall survival (OS), disease specific survival (DSS), disease free interval (DFI) and progression free interval (PFI).

### Tumor microenvironment (TME) analysis

The association between the AR score and TME related factors were analyzed. The R package “ESTIMATE” was used to calculate the stromal, immune, tumor purity, and ESTIMATE score of each patient in the TCGA cohort. The TME-related pathways were obtained and pathway scores were calculated according to the study by Zeng et al. (21). We further analyzed the correlation between AR score and immune cell infiltration with the CIBERSORT algorithm. Immunomodulatory genes, major histocompatibility complex genes, and chemokine/chemokine receptors were also analyzed at pan-cancer level. These correlation results were visualized using heatmaps.

### Statistical analysis

Differences between various groups were compared using Student’s t-test using “ggplot2” or “ggpubr” in R software (https://www.r-project.org/, version:4.1.1). Pearson correlation coefficients were used in correlation analysis. Area under the curves (AUC) of receiver operating characteristic (ROC) was used to assess the predictive accuracy of the AR score. ROC analysis was performed using R package “pROC”, and the AUC values were calculated using auc() function. P < 0.05 (two-tailed) was considered statistically significant.

## Results

### Transcriptional regulation and prognostic value of AR genes in human tumors

**Figure 1:**
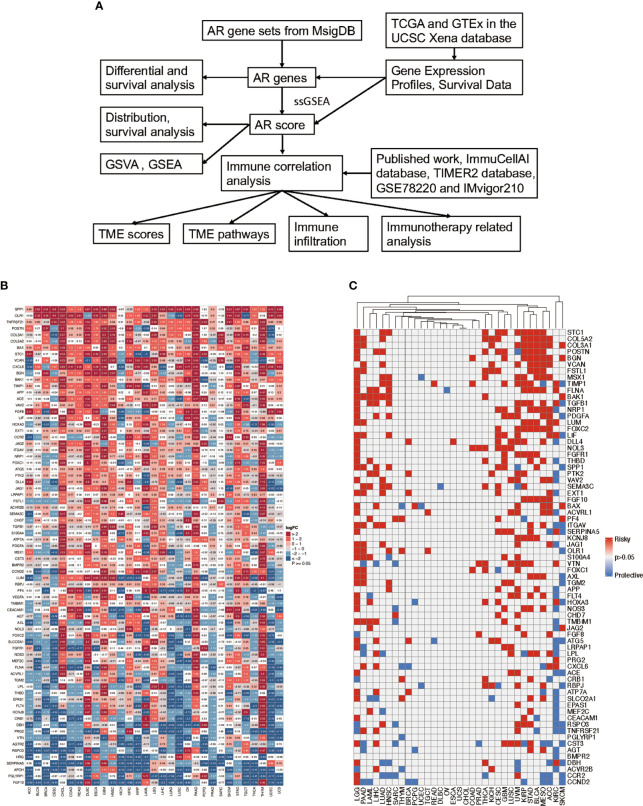
Flow chart of the study and the expression and prognosis of angiogenesis and remodeling (AR) genes. **(A)** Schematic diagram illustrating the research design of this study. **(B)** The heatmap exhibiting the transcriptional level of AR genes in 31 tumor types compared to adjacent normal tissues based on TCGA and GTEx database; the gradient colors represent the log fold change (logFC) value (Red points indicate high expression, while blue points indicate low expression). **(C)** Overall survival analysis of AR genes in 33 tumors of TCGA was analyzed by univariate Cox regression; the gradient colors represent the hazard ratios (Red points indicate high-risk prognostic factor, while blue points indicate protective factor).

The outline of our research is shown in **Figure 1A**. Based on the MsigDB database, 75 AR genes were enrolled in this study, including 36 angiogenic genes and 40 vascular remodeling related genes (**Supplementary Table 1**). Then, we examined their patterns of expression based on TCGA and GTEx database in 31 tumor types. As shown in **Figure 1B**, AR genes were commonly differential expressed in most tumors compared with their corresponding normal tissues, except for Pheochromocytoma and Paraganglioma (PCPG) and Sarcoma (SARC). For example, AR genes were mainly highly expressed in tumors like Pancreatic Cancer (PAAD), Glioblastoma (GBM) and Thymoma (THYM). And taking the complete tumor set as a whole, we noticed that some genes, like *SPP1*, *TNFRSF21* and *BAX*, were usually highly expressed in most tumors, while some in the bottom of this heatmap were mainly in relatively lower expression, represented by *FGF10*. Additionally, we performed uniCox analysis to explore the prognostic value of each gene on OS in 33 tumors (**Figure 1C**). The results indicated that AR gene expression levels affected the survival of cancers, though their impacts may vary depending on tumors. Risk factors, like *STC1*, *COL5A2*, *COL3A1*, and *BGN*, were significantly correlated with poor OS, while *BMPR2* and *PGLYRP1* were only found to be protective factors in 2 of 33 tumor types. There was no predictive gene found in Uterine Carcinosarcoma (UCS) and Cholangiocarcinoma (CHOL).

### The calculation, distribution, and survival analysis of the AR score

**Figure 2:**
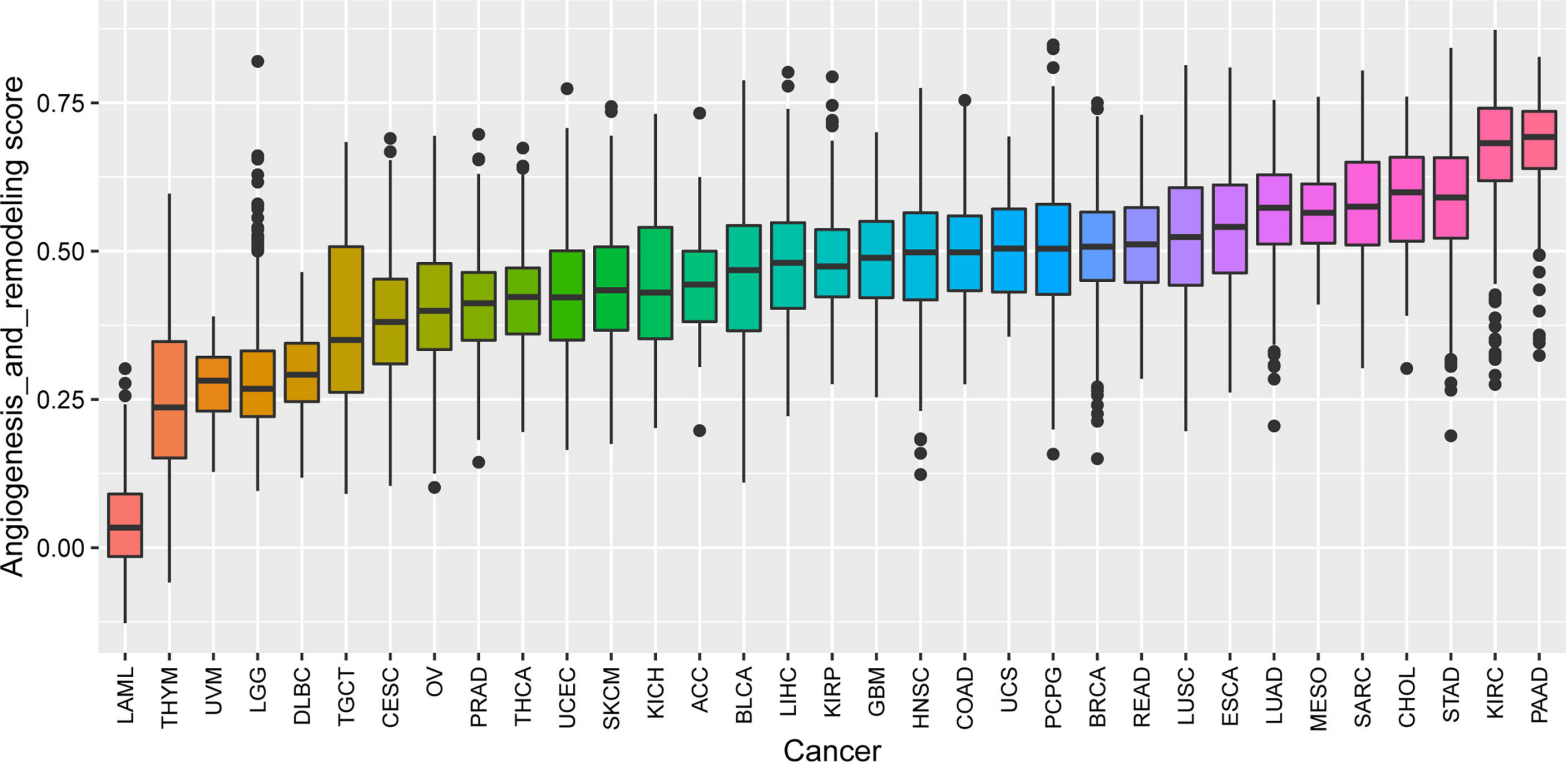
The AR score distribution in different cancer types based on datasets from TCGA. The distribution of the AR score in 33 tumors of TCGA (from low to high). The AR score was highest in Pancreatic Cancer (PAAD) and lowest in Acute Myeloid Leukemia (LAML).

We performed ssGSEA to calculate the AR score across 33 tumor types in the TCGA cohort. A pan-cancer comparison revealed great differences in the AR score among the different types of tumors. The AR score was highest in PAAD and lowest in Acute Myeloid Leukemia (LAML) (**Figure 2**). We also explored the relationship of the AR score with the tumor stage in 26 cancers. The results indicated that the AR score was positively related with stage in some cancers, like Bladder Urothelial Carcinoma (BLCA), Colon adenocarcinoma (COAD) and Stomach adenocarcinoma (STAD). But it was worth noting that the AR score was not associated with the stage in more than half of the tumor types (**Supplementary Figure 1**).

**Figure 3:**
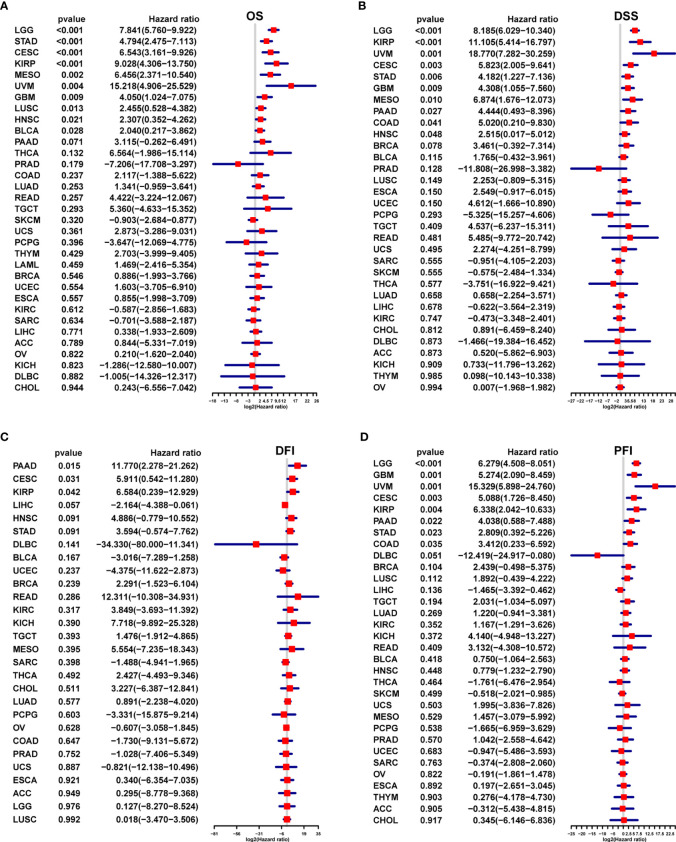
Survival prognostic analysis of the AR score. Univariate Cox regression analysis of the correlation between the AR score and **(A)** overall survival (OS), **(B)** disease specific survival (DFS), **(C)** disease free interval (DFI) and **(D)** progression free interval (PFI) in different tumors.

The results of uniCox analysis indicated that (1): For the OS, AR score was a risk factor in Brain Lower Grade Glioma (LGG), STAD, endocervical adenocarcinoma (CESC), Kidney renal papillary cell carcinoma (KIRP), Mesothelioma (MESO), Uveal Melanoma (UVM), GBM, Lung squamous cell carcinoma (LUSC), Head and Neck squamous cell carcinoma (HNSC), and BLCA (**Figure 3A**) (2). For the DSS, AR score was a risk factor in LGG, KIRP, UVM, CESC, STAD, GBM, MESO, PAAD, COAD, and HNSC (**Figure 3B**) (3). For the DFI, AR score was a risk factor in PAAD, CESC, and KIRP (**Figure 3C**) (4). For the PFI, AR score was a risk factor in LGG, GBM, UVM, CESC, KIRP, PAAD, STAD, and COAD (**Figure 3D**).

**Figure 4:**
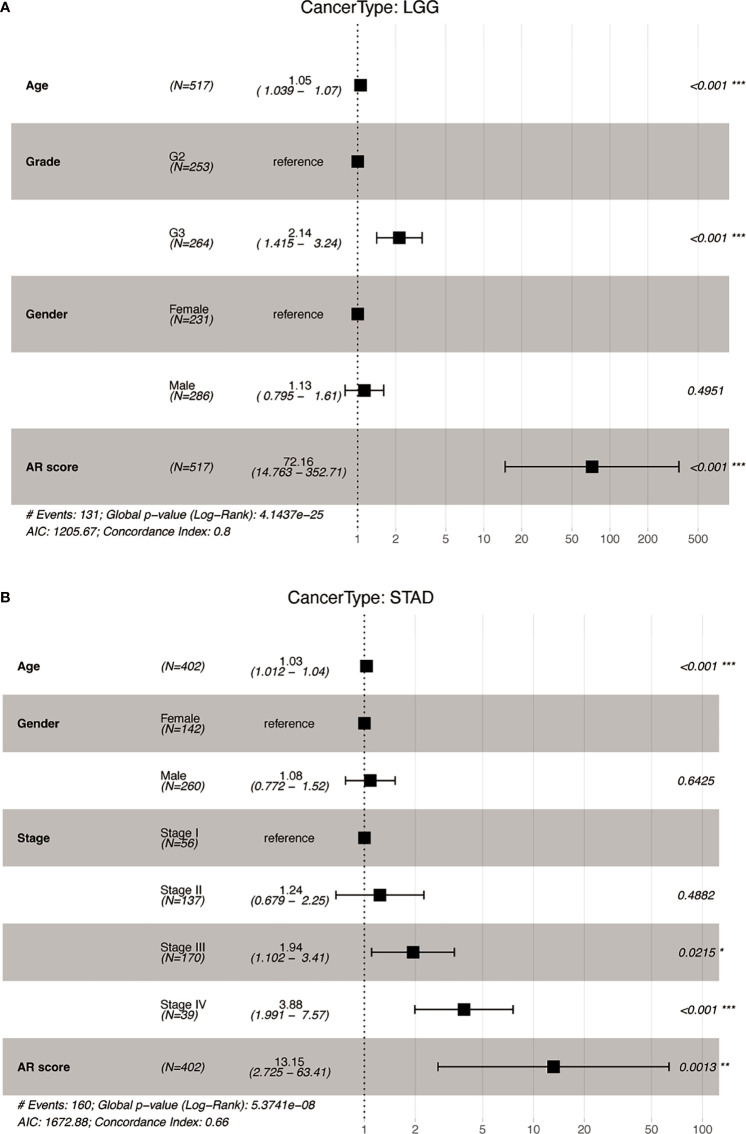
The multivariate Cox analyses of the AR score for OS. Forest map of the AR score and clinicopathological parameters for OS in **(A)** Lower Grade Glioma (LGG) and **(B)** Stomach adenocarcinoma (STAD). *P < 0.05, **P < 0.01, ***P < 0.001.

We further performed multivariant cox regression analysis in LGG and STAD, in which a high AR score was significantly correlated with poor prognosis according to four outcomes (**Figure 3**). After adjusting clinicopathologic data, the AR score was an independent prognostic indicator for OS (**Figure 4**).

### GSVA and GSEA of the AR score

**Figure 5:**
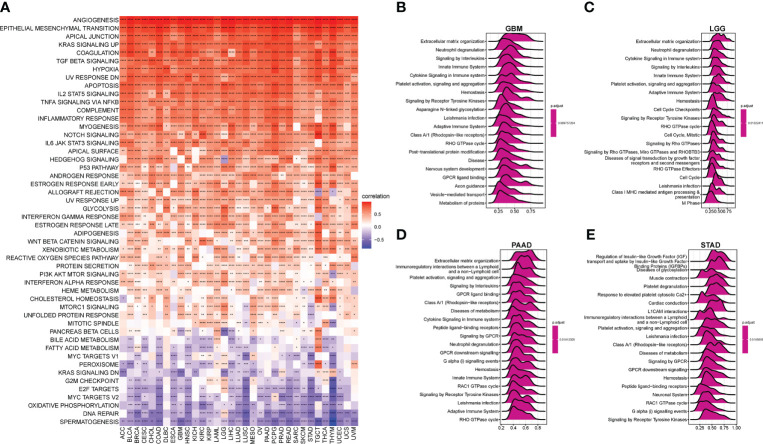
Gene set variation analysis (GSVA) and gene set enrichment analysis (GSEA) of the AR score. **(A)** The heatmap represents the correlation between the AR score and 50 HALLMARK pathways in pan-cancer. And the top 20 significant terms of GSEA results in **(B)** Glioblastoma (GBM), **(C)** LGG, **(D)** PAAD, and **(E)** STAD. *P < 0.05, **P < 0.01, ***P < 0.001, ****P < 0.0001.

To analyze the potential pathways associated with AR, we performed a GSVA and GSEA of the AR score. The association between the AR score and GSVA scores in pan-cancer was shown in **Figure 5A**. We observed that AR score was positively associated with many malignant pathways in pan-cancer, such as epithelial mesenchymal transition, KRAS signaling up, TGF beta signaling, hypoxia, and so on. GSEA results indicated that the AR score was closely associated with extracellular matrix organization pathways and immunoregulatory-related pathways in GBM, LGG, PAAD and STAD (**Figures 5B–E**).

### Correlations between the AR score and TIME in cancers

**Figure 6:**
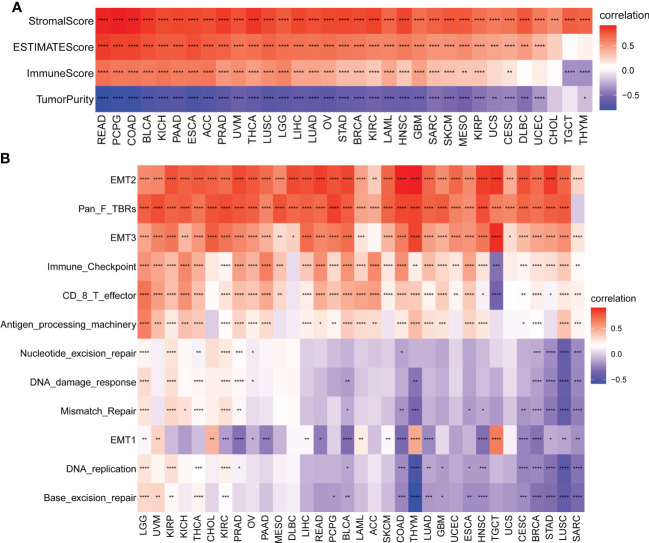
Tumor microenvironment (TME) analysis of AR score. **(A)** Heatmap represents the correlation between the AR score and immune score, stromal score, ESTIMATE score, and tumor purity score in pan-cancer. Red represents positive correlations and blue represent negative correlations. **(B)** Heatmap represents the correlation between the AR score and TME-related pathways. Red represents positive correlations and blue represent negative correlations. *P < 0.05, **P < 0.01, ***P < 0.001, ****P < 0.0001.

Given the importance of TIME in the development of various types of cancers and the correlation between the AR score and immune-related pathways detected previously by GSEA, in a pan-cancer analysis, we also explored the association between the AR score and the estimated proportion of immune and stromal in TIME (**Figure 6A**). The results revealed that the AR score was positively associated with immune score, stromal score, and ESTIMATE score in most cancers. On the contrary, the AR score was negatively correlated with tumor purity. Tumor purity plays a critical role in prediction of prognosis in multiple cancers. Generally, low purity was associated with high immune evasion and poor prognosis (22). Therefore, low AR score might benefit more from immunotherapy.

We further obtained and calculated TME-related pathways according to the published paper (21). The analysis revealed two predominant patterns, depending on the pathways considered (**Figure 6B**): the AR score was positively correlated with immune-related pathways (Immune checkpoint, CD8 T effector, and Antigen processing machinery) and stromal-related pathways (EMT2, Pan F TBRs [TGFB1-related pathways], and EMT3), independently of the type of tumor considered. A different pattern was noticed with the DNA repair-related pathways. The correlations between the AR score and these related pathways were tumor-type independent.

**Figure 7:**
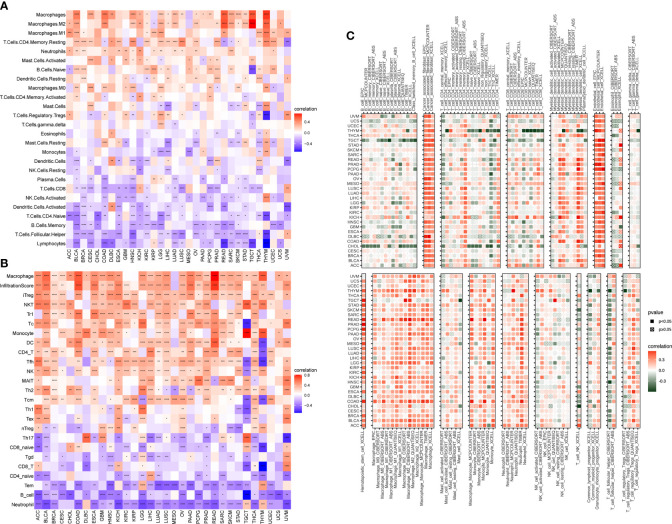
Association of the AR score with immune cell infiltration. Correlation between the AR score and immune cell infiltration based on published work **(A)**, ImmuCellAI database **(B)** and TIMER2 database **(C)**. The red and blue/green colors represent positive and negative correlations, respectively. *P < 0.05, **P < 0.01, ***P < 0.001, ****P < 0.0001.

Moreover, to relate the AR score to immune cells in TIME, we firstly used CIBERSORT algorithm based on a published work (16). As shown in **Figure 7A**, the results revealed that the AR score was positively correlated with infiltration level of TAMs, and negatively associated with natural killer cells, dendritic cells (activated), CD8+ T cells, naive CD4+ T cells, and lymphocytes in pan-cancer (**Figure 7A**). Then, based on the ImmuCellAI (17) and TIMER2 (18) database, the correlation between the AR score and the infiltration of TAMs, CD8+ T cells, naive CD4+ T cells were found repeatedly at pan-cancer level (**Figures 7B–C**). In addition, the AR score was also correlated with other immune cells, like B Cells, Treg, Cancer associated fibroblasts (CAFs), and endothelial cells (**Figures 7B–C**). Additionally, the correlations between the AR score and immune regulation-related genes were explored in more detail in **Supplementary Figure 2**.

### Correlations between the AR score and immunotherapy

**Figure 8:**
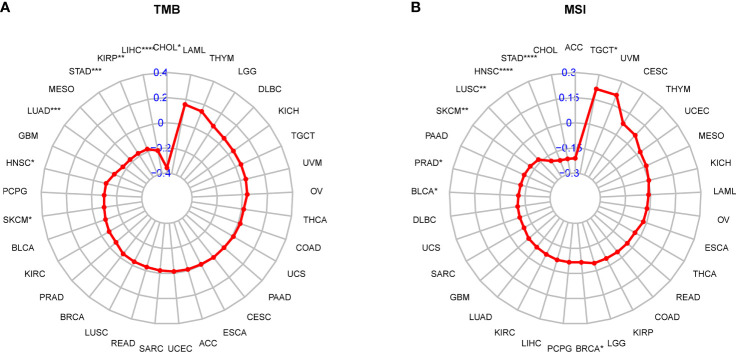
Correlation between the AR score and immunotherapeutic biomarker. **(A)** Radar plot represents the correlation between the AR score and tumor mutation burden (TMB). **(B)** Radar plot represents the correlation between the AR score and microsatellite instability (MSI). *P < 0.05, **P < 0.01, ***P < 0.001, ****P < 0.0001.

Next, considering the results that the AR score was related to immunosuppressive cells and genes, we determined whether AR had a non-ignorable impact on the efficacy of immunotherapy. It was reported that tumor patients with low tumor mutation burden (TMB) or MSI may be resistant to immune checkpoint inhibitors (ICIs) treatment (23–25). Hence, we analyzed the correlation between the AR score and TMB and MSI. As vividly shown in the radar map (**Figure 8A**), the AR score was negatively correlated with TMB in CHOL, Liver hepatocellular carcinoma (LIHC), KIRP, STAD, LUAD, HNSC, and Skin Cutaneous Melanoma (SKCM). In addition, the AR score was positively correlated with MSI in Testicular Germ Cell Tumors (TGCT), but negatively correlated with MSI in BRCA, BLCA, PRAD, SKCM, LUSC, HNSC, and STAD (**Figure 8B**).

**Figure 9:**
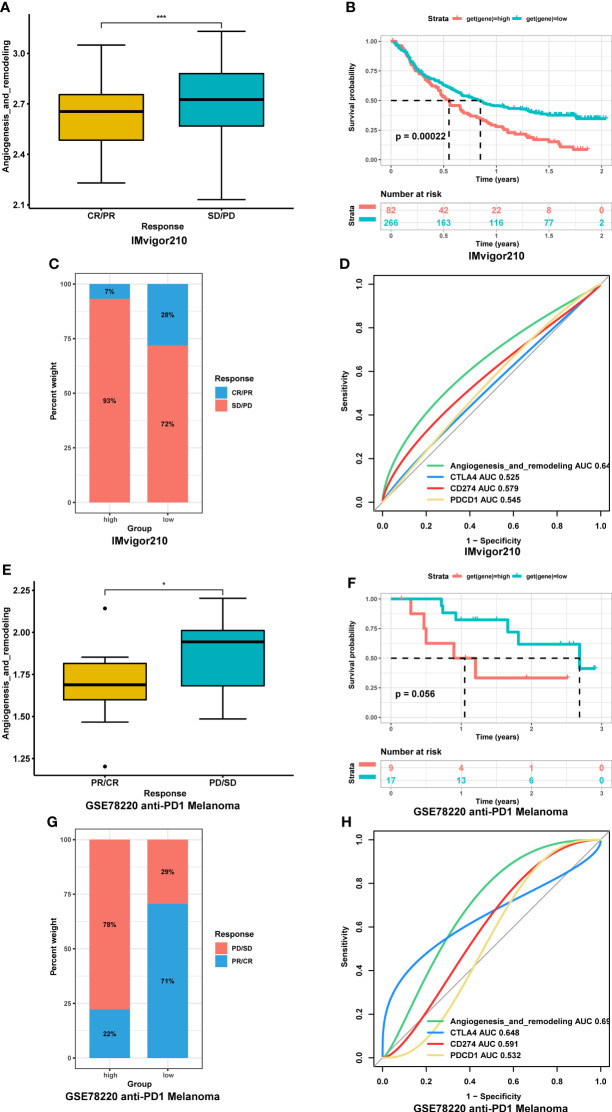
Association between the AR score and immunotherapy response. **(A)** Correction between the AR score and immunotherapy response in IMvigor210 cohort. **(B)** Kaplan-Meier curve of overall survival between high- and low-AR score in IMvigor210 cohort. **(C)** Proportions of complete response/partial response (CR/PR) and stable disease/progressive disease (SD/PD) patients in high- and low-AR score groups in IMvigor210 cohort. **(D)** The receiver operating characteristic (ROC) curve of the AR score and immune checkpoints in IMvigor210. **(E)** Correction between the AR score and immunotherapy response in GSE78220 cohort. **(F)** Kaplan-Meier curve of overall survival between high- and low-AR score in GSE78220 cohort. **(G)** Proportions of CR/PR and SD/PD patients in high- and low-AR score groups in GSE78220 cohort. **(H)** The ROC curve of the AR score and immune checkpoints in GSE78220 cohort. *P < 0.05, ***P < 0.001.

Since the AR score was negatively correlated with TMB and MSI values in most tumor types, it might represent an important new biomarker for ICIs treatment. To link the AR score with the efficiency of immunotherapy, we evaluated the AR score in different response groups based on IMvigor210 bladder cancer cohort. The result showed that the AR score of patients in stable disease/progressive disease (SD/PD) group was significantly higher than that in complete response/partial response (CR/PR) group (**Figure 9A**), and vice versa (**Figure 9C**). In addition, Kaplan-Meier curve was performed in the high and low AR score groups compared with the median level. The result showed that high AR score were significantly correlated with poor OS (P = 0.0002, **Figure 9B**). We also assessed the response data by AUC in different biomarkers, and found that the AR score had a better prediction effect than other immune checkpoints (CLAT4, CD274, and PDCD1) (**Figure 9D**). Moreover, based on GSE78220, similar results were also found in melanomas undergoing ICIs therapy (**Figures 9E–H**).

## Discussion

In present study, we firstly comprehensively explored the landscape of 75 AR-related genes across 33 cancer types. We found great differences in AR gene expression between tumors and adjacent normal tissues in most tumor types, indicating the importance role of AR in in their growth, invasion, and metastasis (26, 27). Besides VEGFA which was widely known and applied in anti-angiogenic therapy, in our pan-cancer analysis, some genes, like *SPP1*, *STC1* and *COL5A2*, were not only highly expressed in most tumors, but also risk factors of prognosis. The comprehending of the function of these genes in AR is crucial for the development of novel biomarker and potential therapeutic target for the anti-angiogenic treatment and against the emergence of drug resistance to VEGF-targeted therapy in advanced cancers (28). We further established a comprehensive AR score based on the ssGSEA method in the pan-cancer analysis. Taking the AR-related genes as a whole, the score varied depending on the types of tumors. And the tumors which scored higher, like KIRC, STAD and LUAD, could benefit from anti-VEGF/VEGFR agents in current clinical practice (29, 30), but also had a poor prognosis. This analysis is open to further studies that may explore the association of the AR score with anti-angiogenetic treatment response.

In agreement with previous studies showing that TIME and infiltrating immune cell subsets could interact with tumor angiogenesis (31–34), we found a correlation between the tumor AR score and immune-related pathways by GSVA and GSEA, for instance, Hypoxia, IL2 STAT5 signaling, signaling by interleukins, innate immune system, and cytokine signaling in immune systems. Then, the AR score was further explored to be positively correlated with proportions and pathways of immune and stromal in TIME in a pan-cancer analysis. And based on the public immune cell infiltration data, we also found that the AR score was positively correlated with the infiltration of immunosuppressive cells, such as TAMs (35) and Tregs (36), but negatively with the tumor-suppressing immune cells in most cancers. These findings are all in line with previous studies that AR-dependent mechanisms promote the generation of an immunosuppressive tumor microenvironment, favoring cancer immune escape and cancer progression (4, 10). These may be the reason for the worse survival status of patients with high AR score. Our study therefore highlights the coordinated interplay between AR and TIME.

The ICIs represent a major advancement in treatment for various types of tumors. There is currently a great need for biomarkers that could predict their efficacy in individuals. The immunosuppressive microenvironment is not conducive to immunotherapy (37–39). Thus, we suspected that patients with high AR score are resistant to immunotherapy. Through analyzing immunotherapy data, we found that patients undergoing ICIs treatment with high AR score had worse therapy efficacy and survival status. In this respect, we provide support to the therapeutic strategy that anti-angiogenic therapies could be considered as putative enhancers of ICIs treatment. As previously mentioned, anti-VEGFRs in combination with ICIs has been tested in RCC, inducing a significantly increase in survival (40, 41). Thus far, there are also promising preclinical and clinical studies ongoing in other cancers, like melanoma, non-small-cell lung carcinoma (28). With the hope that the landscape of tumor AR might be useful for the assessment of the outcome of cancer immunotherapy, further research exploring the contribution of vascular biology to the TIME will improve and open new perspectives on this synergistic efficacy.

However, even though we investigated and integrated information from different databases, there were still some limitations of present study. First, because of the limitation of gene source, we cannot reveal the differentiation of AR-related genes expression comprehensively in the pan-cancer analysis. Clearly, this analysis is open for further studies that may include novel genes of AR as their contribution to TIME. Second, with regard to the shortage of immunotherapy data, we only explore the prediction of AR score in two cancer cohorts. More studies reported about ICIs treatment and related omics data are in great need. When sufficient information has become available, subgroup analyses based on tumor types, ICIs regimens may be conducted in the future. Third, there was a lack of validation of our conclusions in *in vitro* or *in vivo* studies to elucidate the underlying molecular function.

In summary, our study proved that elevated AR score was closely associated with immunosuppressive microenvironment in pan-cancer. Patients with high AR score were resistant to ICIs treatment. And the AR score was a potential biomarker to ICIs treatment in tumor patients.

## Data availability statement

The original contributions presented in the study are included in the article/**Supplementary Material**. Further inquiries can be directed to the corresponding authors.

## Author contributions

XP, YC, and ZL conceived and designed the experiments. XS, ZZ, CY, and HYL obtained the data. XS, ZZ, ZW, RX, TL, and HTL analyzed the data. XS and ZZ wrote the manuscript. RX, YH, XP, YC, and ZL provided advice and edited manuscript. All authors contributed to the article and approved the submitted version.

## Funding

This research was funded by the National Key R&D Program of China (grant No. 2021YFC2500400, 2020YFC2008304), National High Level Hospital Clinical Research (Science and Technology Achievements Transformation Incubation Guidance Fund Project of Peking University First Hospital) (2022CX11), National High Level Hospital Clinical Research Funding (Interdepartmental Clinical Research Project of Peking University First Hospital) (no. 2022CR36), and National Natural Science Foundation of China (grant No. 81973320 and No. 81903714).

## Conflict of interest

The authors declare that the research was conducted in the absence of any commercial or financial relationships that could be construed as a potential conflict of interest.

## Publisher’s note

All claims expressed in this article are solely those of the authors and do not necessarily represent those of their affiliated organizations, or those of the publisher, the editors and the reviewers. Any product that may be evaluated in this article, or claim that may be made by its manufacturer, is not guaranteed or endorsed by the publisher.
