# Supplementary material for: The role of Angiogenesis and remodeling (AR) associated signature for predicting prognosis and clinical outcome of immunotherapy in pan-cancer
Source: Front Immunol. 2022 Nov 21;13:1033967. doi: 10.3389/fimmu.2022.1033967 (PMC9719961; doi:10.3389/fimmu.2022.1033967)
Supplement: Supplementary file 1 [file Table_1.docx]

Supplementary Table 1

| The list of the angiogenesis and remodeling genes |
| --- |
| VCAN; POSTN; FSTL1;LRPAP1; STC1;LPL; VEGFA; PF4; THBD; FGFR1; TNFRSF21; CCND2; COL5A2; ITGAV; SERPINA5; KCNJ8; APP; JAG1; COL3A1; SPP1; NRP1; OLR1; PDGFA; PTK2; SLCO2A1; PGLYRP1; VAV2; S100A4; MSX1; VTN; TIMP1; APOH; PRG2; JAG2; LUM; CXCL6; SEMA3C; CST3; DBH; ACE; AGT; AGTR2; EPAS1; EXT1; FGF8; FGF10; FOXC1; FOXC2; FLNA; FLT4; CRB1 HOXA3; HRG; RBPJ; LIF; MEF2C; NOS3; ATP7A; DLL4; CHD7; AXL; BAK1; BAX; BGN; CEACAM1; TMBIM1; BMPR2; TGFB1; TGM2; CCR2; RSPO3;NOL3; ACVR2B; ACVRL1; ATG5 |
