# Supplementary figures and images for: The role of Angiogenesis and remodeling (AR) associated signature for predicting prognosis and clinical outcome of immunotherapy in pan-cancer
Source: Front Immunol. 2022 Nov 21;13:1033967. doi: 10.3389/fimmu.2022.1033967 (PMC9719961; doi:10.3389/fimmu.2022.1033967)

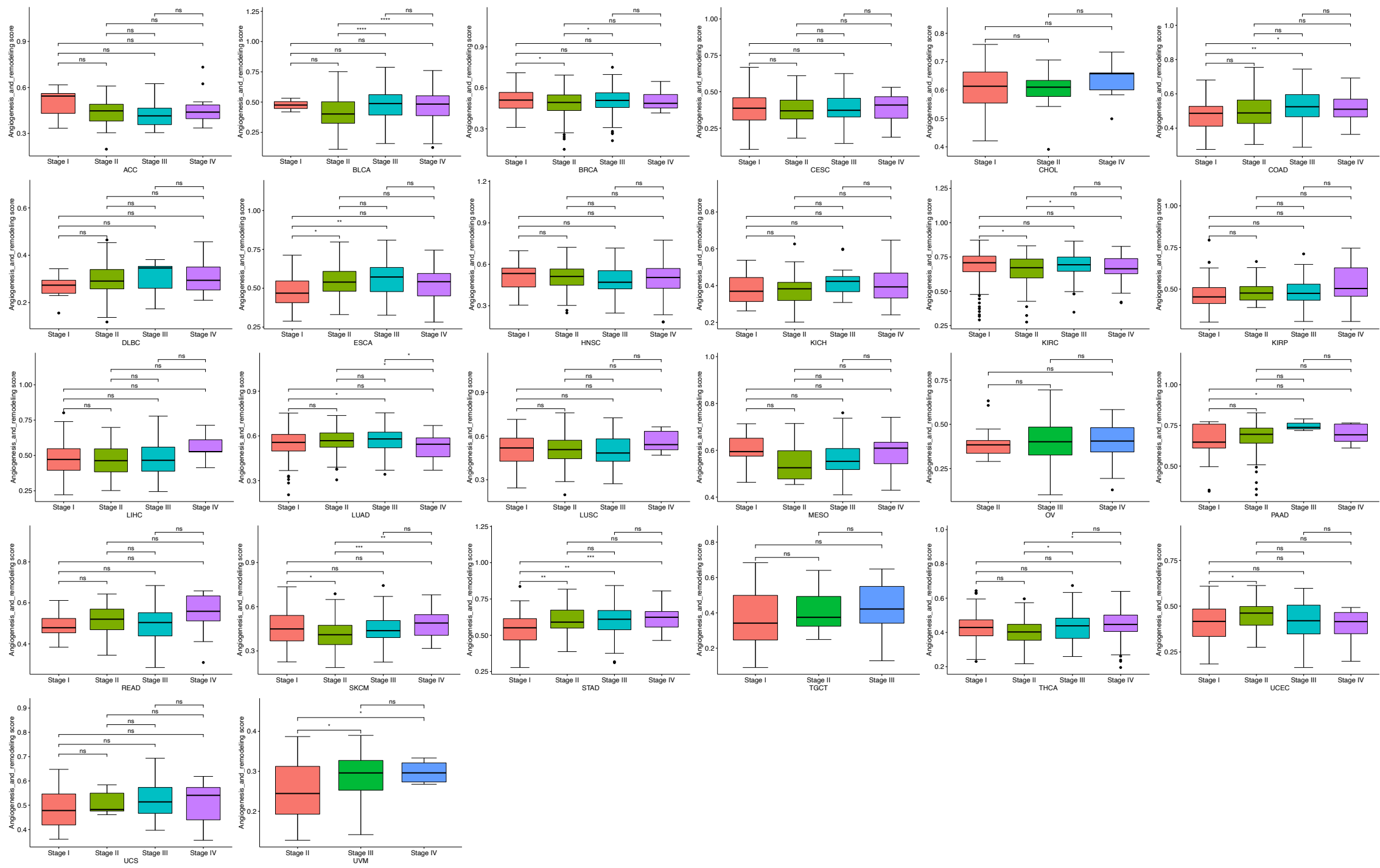

Supplement: Supplementary file 2 [file Image_1.pdf]

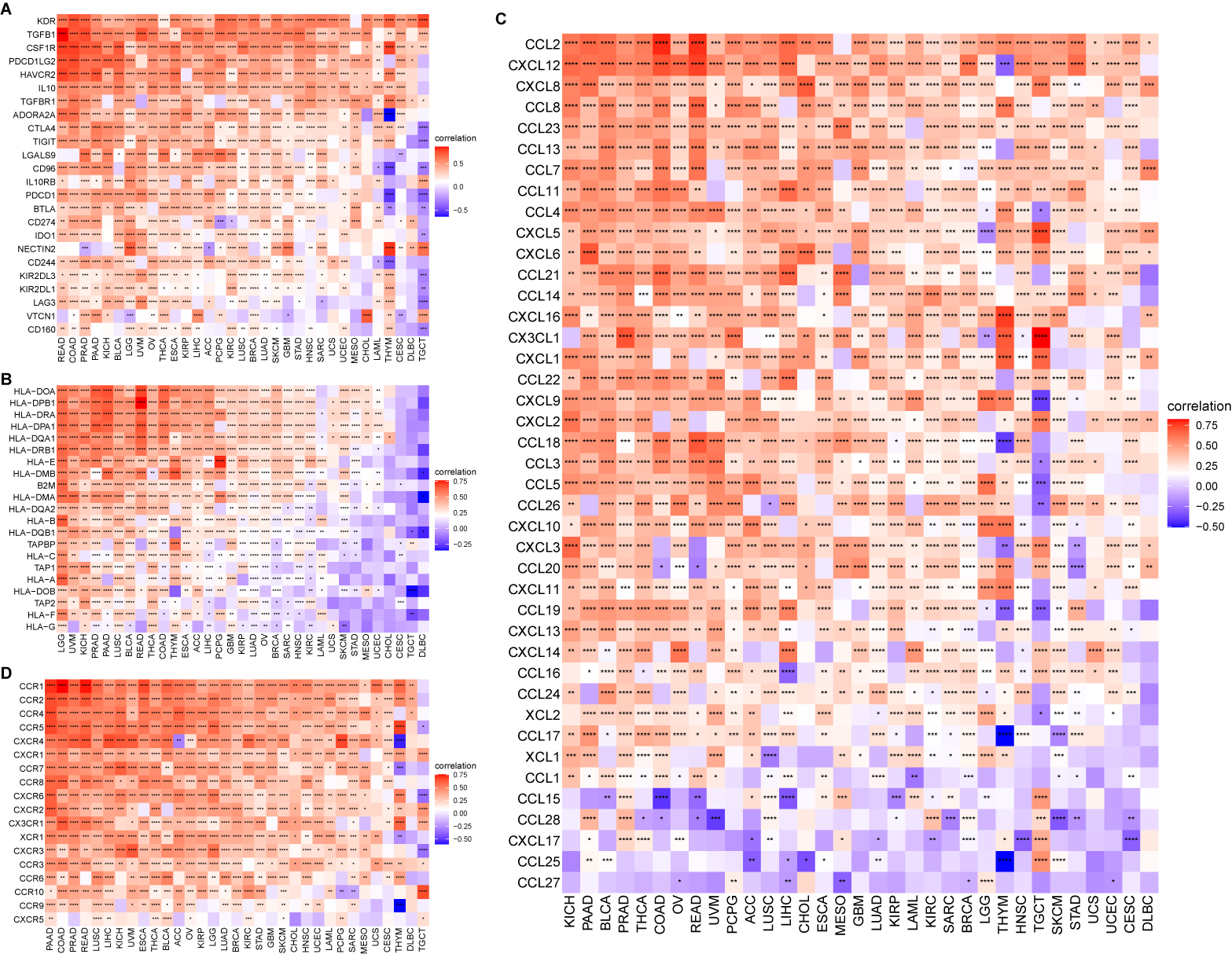

Supplement: Supplementary file 3 [file Image_2.tif]
